# Forecasting cryptocurrency's buy signal with a bagged tree learning approach to enhance purchase decisions

**DOI:** 10.3389/fdata.2024.1369895

**Published:** 2024-05-09

**Authors:** Raed Alsini, Qasem Abu Al-Haija, Abdulaziz A. Alsulami, Badraddin Alturki, Abdulaziz A. Alqurashi, Mouhamad D. Mashat, Ali Alqahtani, Nawaf Alhebaishi

**Affiliations:** ^1^Department of Information Systems, Faculty of Computing and Information Technology, King Abdulaziz University, Jeddah, Saudi Arabia; ^2^Department of Cybersecurity, Faculty of Computer and Information Technology, Jordan University of Science and Technology, Irbid, Jordan; ^3^Department of Information Technology, Faculty of Computing and Information Technology, King Abdulaziz University, Jeddah, Saudi Arabia; ^4^Department of Computer Science, Faculty of Computing and Information Technology, King Abdulaziz University, Jeddah, Saudi Arabia; ^5^Department of Networks and Communications Engineering, College of Computer Science and Information Systems, Najran University, Najran, Saudi Arabia

**Keywords:** trading strategies, machine learning, technical indicator, cryptocurrency market, data-driven trading

## Abstract

**Introduction:**

The cryptocurrency market is captivating the attention of both retail and institutional investors. While this highly volatile market offers investors substantial profit opportunities, it also entails risks due to its sensitivity to speculative news and the erratic behavior of major investors, both of which can provoke unexpected price fluctuations.

**Methods:**

In this study, we contend that extreme and sudden price changes and atypical patterns might compromise the performance of technical signals utilized as the basis for feature extraction in a machine learning-based trading system by either augmenting or diminishing the model's generalization capability. To address this issue, this research uses a bagged tree (BT) model to forecast the buy signal for the cryptocurrency market. To achieve this, traders must acquire knowledge about the cryptocurrency market and modify their strategies accordingly.

**Results and discussion:**

To make an informed decision, we depended on the most prevalently utilized oscillators, namely, the buy signal in the cryptocurrency market, comprising the Relative Strength Index (RSI), Bollinger Bands (BB), and the Moving Average Convergence/Divergence (MACD) indicator. Also, the research evaluates how accurately a model can predict the performance of different cryptocurrencies such as Bitcoin (BTC), Ethereum (ETH), Cardano (ADA), and Binance Coin (BNB). Furthermore, the efficacy of the most popular machine learning model in precisely forecasting outcomes within the cryptocurrency market is examined. Notably, predicting buy signal values using a BT model provides promising results.

## 1 Introduction

Cryptocurrencies have achieved worldwide recognition and are familiar to most individuals due to the rapid development of e-commerce, the financial industry, and blockchain technology. Through the Internet, the cryptocurrency market has attracted the attention of investors (Matytsin, 2021). Throughout history, cash has consistently been one of the most popular payment methods for settling any deal. Credit cards and personal checks have become widespread ways to pay for in-store and online purchases. Payment systems have continuously developed to meet the requirements of new technologies (Saxena et al., 2019). Cryptocurrency is a new approach to a digital currency payment system that has been developed to date, thanks to advances in finance known as FinTech (Vo and Yost-Bremm, 2018). Cryptocurrency is a digital currency designed to make online financial transactions more secure and private by utilizing a peer-to-peer network protocol and decentralized by applying blockchain technology.

**Figure 1:**
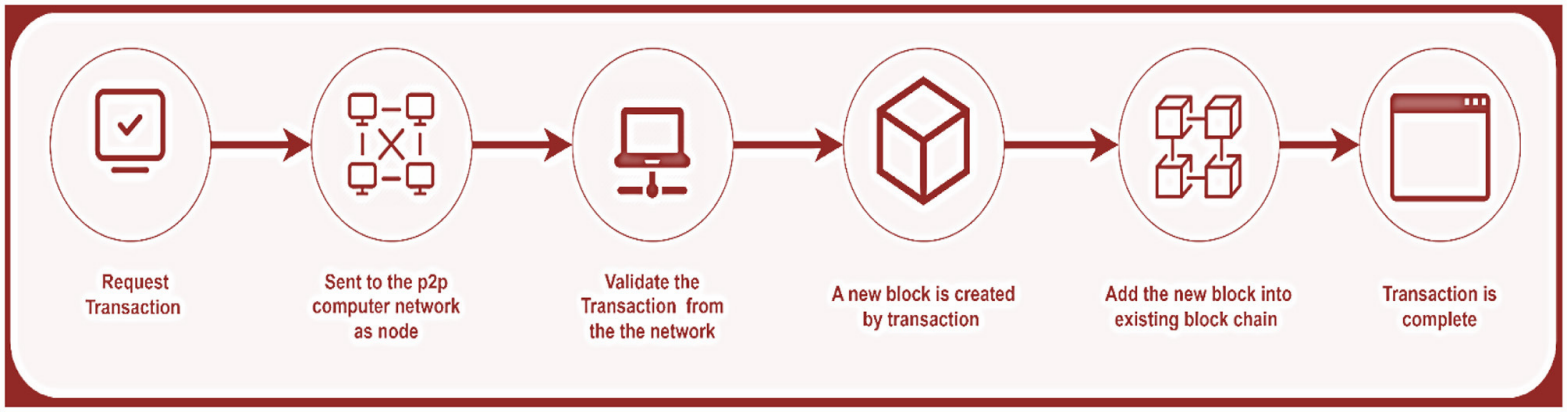
Framework of blockchain.

A Blockchain can be defined as “a chain of blocks.” The block refers to a grouping of transactions in the distributed ledger. Every information related to a specific transaction is stored in a block (Namasudra et al., 2021). The sender and the receiver are labeled, as are the date, time, and the total amount being transferred, as shown in Figure 1. As the name implies, a chain is an unbroken sequence of blocks that allows anyone to trace the complete transaction history of a given asset back to its inception (Senthilkumar, 2021).

There has been much attention focused on cryptocurrencies recently. Nakamoto (2017) introduced Bitcoin in 2008 as a decentralized digital currency that does not rely on a central bank or any other third party to transfer funds between users over the Internet. Bitcoin and other cryptocurrencies like it were designed to eliminate the need for and the associated fees charged by financial institutions. Additionally, because the currency is digital, there is no upper limit on how much can be made. In addition, cryptocurrencies are a global financial asset that can be accessed from various locations worldwide because they are not tied to any one central or regional authority and are remarkably accessible to use (Cunha and Sebastião, 2021).

Forecasting Bitcoin prices is crucial for asset managers and individual investors. Unlike traditional currencies, Bitcoin's unique characteristics, like transaction speed, decentralization, and a large community of enthusiasts, make it challenging to apply standard economic theories related to supply and demand dynamics (Lamothe-Fernández et al., 2020). No government or central bank backs Bitcoin and other cryptocurrencies. As a result, their worth is largely dependent on popular opinion and how they are perceived as assets (Lee, 2019). Social media users willingly divulge their opinions and feelings, which machine learning algorithms can use to foretell the future value of cryptocurrencies (Patel et al., 2020; Vachhani et al., 2020; Iqbal et al., 2021). A wide range of scholars has investigated several methods of commodity trading. However, very little study has been done on the algorithmic trading of cryptocurrencies with solutions that integrate trading and tackle high market volatility (Hairudin et al., 2022).

Algorithmic trading has become very popular among investors since the development of machine learning (ML) algorithms. “algorithmic trading” refers to using machine learning to make trades automatically on financial markets (Cohen, 2022). An interesting area of study is using machine learning in the cryptocurrency market. First, there are exchanges where people may buy and sell cryptocurrencies, like Bitcoin, for conventional currencies. However, major limitations to Bitcoin still make it hard to buy and sell products and services (Matytsin, 2021). That's Why it's important for algorithmic trading in cryptocurrencies to function similarly to machine learning outcomes.

The financial market employs another form of analysis known as technical analysis to solve the issue arising in the algorithmic trading of cryptocurrencies, which differs from fundamental analysis (Shah et al., 2019; Fang et al., 2022). Because of its fundamental assumptions, technical analysis employs a variety of approaches and mechanisms (Anghel, 2021). In a technical analysis, price data for an asset is assumed to reflect supply and demand. This way, market participants can anticipate price movements by looking at historical price data (Vo et al., 2019). Technical analysis states that traders use various indicators to monitor price patterns. Based on this explanation, it's clear that the technical analysis approach is gaining popularity due to its accessibility. Technical analysis has become increasingly popular as a trading strategy because it is simple to learn and implement daily (Detzel et al., 2021).

Most research on technical analysis indicators has been done in the context of the stock, forex, and futures markets. It has typically only compared benchmark performances based on the expected net profit at the end of the investment period. The Relative Strength Index (Gurrib and Kamalov, 2019), the Moving Average Convergence/Contraction (Cohen and Qadan, 2022), the On-Balance-Volume, and the Price Trends indicators were used to analyze the data. Bitcoin (BTC), Ethereum (ETH), and XRP (XRP) all have price information from various periods. Three different cryptocurrencies were measured the same way using these indicators in terms of performance after technical analysis methods were applied to them (Çelik, 2019). Fixed parameters are used by comparing the indicators obtained with fixed parameters and those obtained with variable parameters in terms of the reliability of the signals produced and the sustainability of these strategies.

By taking this need into account, we aim to analyze the effect of technical analysis on the buy signal using ML models of the following indicators: Bollinger band (BB), Moving Average convergence/divergence (MACD), and Relative Strength Index (RSI).

Studying the buy signal is the main focus of this research because it is a crucial part of making the right decision when buying cryptocurrency. Meanwhile, Dollar Cost Averaging (DCA) is a method for minimizing the impact of volatility on cryptocurrency investments by purchasing the asset regularly. Enhancing purchasing decisions can help improve the outcomes of the DCA strategy.

The summary of the contributions in this research is as follows:

- Collecting real-time cryptocurrency data from the Binance exchange.
- Computing technical indicators using the raw data collected from the Binance exchange.
- Exploring the efficacy of the buy signal from each technical indicator in anticipating accurate purchase decisions.
- Utilizing an ensemble model, i.e., Bagged Tree (BT), to predict the buy signal.

The paper's organization is as follows: The relevant research is presented in Section 2. Section 3 describes the methods used to identify predictability patterns. Section 4 presents the results of our study and a discussion. Section 5 summarizes our findings and suggests future research opportunities for enhancing our model.

## 2 Related work

The development of cryptocurrencies, assets that combine cutting-edge technology with financial innovation, has displayed the digitization of finance. As the Bitcoin market grows, it has become a focus point for its tremendous return potential and vulnerability to market manipulation. This part of the literature reviews the growing amount of research to understand and reduce the hazards caused by such manipulative practices and predict prices with frequent price changes in the market. The review follows the evolution of machine learning applications in identifying and forecasting market prices, beginning with models launched in 2021 and progressing to real-time detection systems produced in 2023.

Jaquart et al. (2021) used machine learning models, including GRU, LSTM, and others, to make short-term forecasts for the Bitcoin market. The purpose was to evaluate these models' ability to anticipate market changes. A study by Asgari and Khasteh (2021) concentrated on developing trading techniques for cryptocurrencies such as Ethereum and others using machine learning models such as KNN and Gradient Boosting. The study aimed to devise effective trading tactics for the unpredictable cryptocurrency market. The article (Fang et al., 2021) investigated the application of LSTM neural networks to estimate price formation in cryptocurrency marketplaces with a high accuracy rate, concentrating on Bitcoin's mid-price swings.

Yu (2022) investigated using several machine learning models, such as Random Forest and LSTM, to build trading techniques in the Bitcoin market. A wide variety of market data was used to evaluate the models. The research by Bellocca et al. (2022) looked at the prevalence and direction of overreaction circumstances in the Bitcoin, Ethereum, and Litecoin markets. Many machine learning models were applied to analyze these market behaviors. Dolatsara et al. (2022) used a classifier and regression tree model to create an understandable decision support system for daily Bitcoin trading, with an outstanding accuracy of 98.59%. Researchers in Toledo and Souza (2022) forecasted trading signals in multiple cryptocurrencies, emphasizing closing price behavior. They employed models such as Logistic Regression, LightGBM, and PCA for enhanced analysis. The work by Arowolo et al. (2022) used ICA-Firefly Linear-SVM and ICA-Firefly-SigmoidSVM to create a high-accuracy prediction model for Bitcoin prices, demonstrating the possibility for real-time use.

La Morgia et al. (2023) used Random Forest and AdaBoost to build a machine-learning model to detect pump and dump instances in the DogeCoin and Ripple markets, analyzing many such events to highlight the dangers of market manipulation. Hu et al. (2023), a neural network model known as a sequence-based neural network (SNN) was introduced to predict the likelihood of pump-and-dump behavior in Bitcoin trading. To improve detection precision, this model employs a positional attention mechanism. Researchers from Bello et al. (2023) created an LSTM-based auto-encoder for their Low Latency Detection system, which quickly identifies pump and dump activity in the Bitcoin market – facilitating speedier implementation of trade suspension methods.

Shifting the focus to profitability, Liu et al. (2023) introduced a novel method for predicting hospitality order cancellations that maximize business gains. This study proposes a new method to predict and prevent cancellations in hospitality bookings, ultimately increasing profits. They developed a unique profit-focused model that identifies high-risk cancellations and has been proven effective through real-world data and sensitivity analysis. The approach goes beyond hospitality and can be applied for profit-driven prediction in other industries. Hotel managers can directly benefit by using this method to optimize revenue management. In the same context, Jiang et al. (2024) proposed a new method to predict customer churn that focuses on maximizing profits. It combines multiple profit-driven models and uses a special algorithm (inspired by hummingbirds) to find the best way to weight them for the most profit. The method is also designed to be understandable, using an approach to explain why specific customers are predicted to churn. Tested on real-world data across various industries, this method outperforms existing ones in terms of profit gain while remaining interpretable. Finally, Table 1 summarizes the comparison of the performance of existing works.

**Table 1:** Comparison of performance of existing works.

**Article**	**Year**	**Model(s)**	**Cryptocurrency focus**	**Key findings**	**Accuracy**
Jaquart et al. (2021)	2021	GRU, LSTM, FNN, LR, RF, Ensemble, RNN	BTC	Machine learning is utilized to predict short-term market movements across various models.	50.9%−56%
Asgari and Khasteh (2021)	2021	KNN, RF, eXtreme Gradient Boosting,	ETHUSDT, LTCBTC, ZECBTC	Machine learning is used to create trading strategies for cryptocurrency markets.	ETHUSDT: 51.9%−56.3%, LTCBTC: 58.5%−52.05%, ZECBTC: 52.1%−51.86%
Fang et al. (2021)	2021	NN (LSTM)	BTC	Their goal is to determine price formation in Bitcoin marketplaces.	86%
Yu (2022)	2022	RF, XG Boost, AdaBoost, Light GBM, LSTM	BTC	We investigated several machine-learning models for Bitcoin trading strategies.	RF: 68%, AdaBoost: 69%, Light GBM: 70.4%, XG Boost: 70.28%, LSTM: 68%
Bellocca et al. (2022)	2022	SVM, GNB, MNB, KNN, LG, RFC, MLP	BTC, ETH, LTC	Various models were used to investigate the existence and guidance of overreaction situations.	BTC: 67%−77%, ETH: 68%−74%, LTC: 71%−78%
Dolatsara et al. (2022)	2022	Classifier and regression tree	BTC	Created an understandable decision support system for everyday Bitcoin trading.	98.59%
Toledo and Souza (2022)	2022	LR, LR-PCA, LightGBM, LightGBM-PCA, XGBoost, XGBoost-PCA	BTC, ETH, BNB, ADA, XRP	Signal prediction in bitcoin trading activities, emphasizing near price behavior.	BTC: 51%−54%, ETH: 53%−43%, BNB: 45%−54%, ADA: 52%−48%, XRP: 49%−50%
Arowolo et al. (2022)	2022	ICA-FireflyL-SVM, ICAFirefly-SigmoidSVM	BTC	Created a model that predicts BTC values with excellent accuracy.	95%, 96.67%
La Morgia et al. (2023)	2023	Random Forest and Adaboost	DogeCoin (DOGE) and Ripple (XRP)	A machine learning model was created to recognize pump and dump events. Over 900 incidents were examined, exposing market manipulation hazards.	RF: 94.5%, AdaBoost: 93.1%
Hu et al. (2023)	2023	LR, RF, SNN	BTC	Based on 709 events, they created an SNN to forecast pump probabilities. Positional attention was used to improve the detection of pump-and-dump systems.	Pump message detection: LR: 90.2%, RF: 92%; Target coin prediction: Up to SNN: 79.7%
Bello et al. (2023)	2023	LSTM	BTC	We have developed a low latency detection system based on an LSTM-based auto-encoder for identifying pump and dump actions capable of fast detection to help with trade suspension.	LSTM: 80%

## 3 Methodology and dataset

### 3.1 Collecting the dataset

The decentralized nature of cryptocurrencies makes it difficult to collect data from them for research purposes. However, we overcome it by gathering our datasets using two separate methods. The Crypto Currency Exchange Trading Library (CCXT) was our first point to reach out to, as it offered an integrated API that could retrieve data from several exchanges and was frequently used (BTC/USDT, 2022). For example, Binance - a prominent Bitcoin exchange – gave us raw statistics (CCXT/CCXT, n.d.). Today's four biggest traded cryptocurrencies—BTC, ETH, ADA, and BNB—were the focus of our analysis (Cryptocurrency Exchange for Bitcoin, Ethereum, Altcoins, and Binance, 2023). The data was obtained from November 2018 to November 2021. With rows indicating 15-min intervals, the price dataset for each digital currency showed how much each coin's value fluctuated. Among the many attributes included in this collection were timestamps for each transaction, open prices, high/low/close data, and trading volumes.

Next, we collected cryptocurrency price data and applied it to develop technical indicators using a Python model to process the data collection. To evaluate market movements and develop trading signals, we use TA-LiB as a technical analysis library supporting statistical tools (Hansen et al., 2022). Then, we trained our model using the library to extract the technical signal. Moving averages, Relative strength index (RSI), Moving average convergence divergence (MACD), and Bollinger bands (BB) were part of the analytical tools.

### 3.2 Cryptocurrency selection

Due to its unique opportunity for investment and massive profit potential, the cryptocurrency area has attracted significant attention in the past several years. Cryptocurrencies are traded on many exchanges, where participants buy and sell actions based on market fluctuations and news updates. Bitcoin (BTC), Ethereum (ETH), Binance Coin (BNB), Cardano (ADA), and others are among the most well-known cryptocurrencies (Ta-lib/Ta-lib-Python, n.d.). More details about how to choose those coins are discussed below:

1. **Bitcoin**: Regarding market value and popularity, Bitcoin is the earliest and largest cryptocurrency. It is a decentralized digital currency that enables peer-to-peer transactions without intermediaries. Bitcoin is exchanged on numerous cryptocurrency exchanges, with supply and demand dynamics determining its price. The extreme volatility of Bitcoin creates both possibilities and problems for traders, and numerous trading tactics, including technical analysis and algorithmic trading, have been presented (Smales, 2022). Bitcoin's popularity has resulted in the introduction of other Bitcoin-related financial instruments, including futures and options (Joiner et al., 2022).
2. **Ethereum** is a decentralized blockchain platform that enables the development of smart contracts and decentralized applications. It has its own money, Ether, used to pay for Ethereum network transactions. Interest in Ethereum has been high due to its support for non-fungible tokens (NFTs) and its ability to facilitate decentralized financial applications. Ethereum is listed on several cryptocurrency exchanges, where demand and adoption trends impact its value. A few methods to trade Ethereum include using technical analysis and keeping focused on patterns (Shynkevich, 2021).
3. **Cardano** is a blockchain platform that uses the proof-of-stake consensus technique to build a secure and scalable platform for decentralized applications. Additional aspects of Cardano include ADA, a digital currency used to pay fees and transactions on the Cardano network. Cardano is a prominent cryptocurrency that aims to facilitate decentralized financial applications through its dedication to peer-reviewed development, thorough scientific research, and stringent scientific standards. Cardano is listed on multiple cryptocurrency exchanges, and market movements and the adoption rate influence its price. Cardano trading approaches entail conducting technical analysis and employing trend-following strategies (Fang et al., 2022).
4. **Binance Coin** (BNB) is a platform token issued by the Binance Exchange. It is built on the Ethereum blockchain, complies with the ERC20 standard (Busayatananphon and Boonchieng, 2022), and reaches a maximum supply of 200 million tokens (Sun and Yu, 2020; Kumar and Rajesh, 2022).

The dataset synthesizes the previously discussed cryptocurrencies Bitcoin, Ethereum, Cardano (ADA), and Binance Coin (BNB). Table 2 thoroughly examines strategic purchasing decisions, utilizing three notable technical indicators: BB, RSI, and MACD buy signals. In the provided table, each buy signal is used to identify the transaction records associated with either no buying label or buying label. The data collected over a given time reveals a significant occurrence of no buying labels, which can be attributed to the current market volatility and the cautious position adopted by investors. Also, the data reveals the difference in the percentage of buy signals between the three indicators. The cryptocurrency market comprises a complicated interaction between investor sentiment, market manipulation strategies, legislative changes, and technological progress (Binance Exchange, 2018). Emotional factors, like fear and the fear of missing out (FOMO), can trigger unexpected price shifts.

**Table 2:** Correlation between technical buy signals and transaction records.

**Buy signal**	**Transaction record**		
	**Not to buy**	**Buy**	**Buy signals (%)**
Bollinger bands (BB)	3,57,882	61,696	14.70%
Relative strength index (RSI)	4,04,404	15,174	3.61%
Moving average convergence divergence (MACD)	2,11,046	2,08,532	49.70%

Table 3 presents a detailed summary of different features, providing an exhaustive analysis of the dataset used for simulating outcomes. These characteristics include a variety of properties, such as timestamps, which identify the exact periods important to the trading model. Another notable feature is using numerical values to indicate crucial aspects of the bitcoin market price. These characteristics include the opening, high, low, close, and volume that presented the market candlestick. In addition, it contains the calculated RSI, which presents the decision to buy and sell. MACD, MACD_signal, and Machinist values are all features used to identify any change in the trading price. The Bollinger Bands (BB) purchase signal, which includes the upper, middle, and lower bands, is also presented numerically. The BUY/SELLprice_BB, BUY/SELLprice_RSI, and BUY/SELLprice_MACD are calculated and used in the dataset. The buy signal employs a binary toggle function to calculate the overall number of purchases logged.

**Table 3:** Features name and attributes used in the dataset.

**Feature**	**Attribute**
Time	Time Stamp
Open	Numerical
Hight	Numerical
Low	Numerical
Close	Numerical
Volume	Numerical
RSI	Numerical
MACD	Numerical
MACD_signal	Numerical
Machinist	Numerical
BB_upper	Numerical
BB_middle	Numerical
BB_lower	Numerical
BUY/SELLprice_BB	Numerical
BUY/SELLprice_RSI	Numerical
BUY/SELLprice_ MACD	Numerical
BB_buy_signal	Binary Toggle
RSI_buy_signal	Binary Toggle
MACD_buy_signal	Binary Toggle

### 3.3 Crypto-market strategy

Crypto market strategy refers to the method or approach traders and investors employ to make lucrative transactions in the cryptocurrency market. In layperson's terms, it determines when to purchase, sell, or hold a certain cryptocurrency. Because the cryptocurrency market is volatile, it is critical to establish a solid market strategy based on various elements such as technical analysis, fundamental analysis, market trends, and risk management.

Recently, a study was done on effective tactics in the Bitcoin market. Wilder (1978) analyzed the success rates of various trading methods, such as buy-and-hold strategies, moving average crossovers, and momentum-based approaches within cryptocurrency markets. The study states that momentum-driven approaches provide higher return alternatives, regardless of market conditions. Another approach is applying machine learning models to understand the cryptocurrency trading system better, as shown in Kamara et al. (2022). Their approach relied heavily on technical indicators and used ML, enabling them to predict trends accurately.

### 3.4 Technical analysis indicator

In modern financial markets, Investors and traders apply techniques, such as technical analysis, to track the movement of price fluctuations and identify patterns to make the right decision. The Relative Strength Index (RSI), Bollinger Bands, and Moving Average Convergence Divergence (MACD) are promising buying indicators and highlight significant market trends.

#### 3.4.1 Relative strength index

The relative strength index (RSI) is an indicator that most cryptocurrency exchanges have as a standard feature (Lauguico et al., 2019). It determines if prices increase or decrease at a certain rate and is classified as a momentum oscillator.


$$\begin{array}{l} RSI\;=\;100\;\left(\frac{100}{\left(1+\;RS\right)}\;\right) \end{array}$$

**Figure 2:**
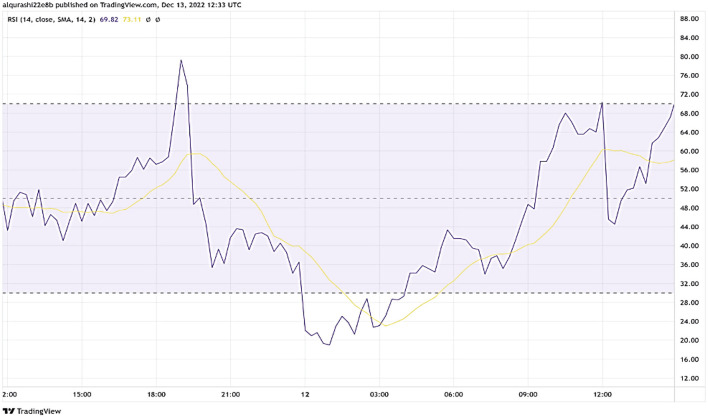
Buy signal = 1 if the RSI value < 30.

The measure of relative strength, or RS, is the average of positive and negative closing values for a certain period divided by the average of those two variables. The typical range for the Relative Strength Index (RSI) is from zero to one hundred. Most people consider values above 70 to be overbought, suggesting a possible correction or reversal in the market. A market recovery is likely when values drop below 30, known as oversold. Using the following formula, one may find the RSI value for a 14-period RSI by averaging the gain and loss from the previous and current periods (illustrated in Figure 2):


$$\begin{array}{l} RSI\;=\;100\;-\;\left[\frac{100}{1\;+\left(\frac{\left(Previous\;Average\;Gain\times 13\right)+\;Current\;Gain}{\left(Previous\;Average\;Loss\times 13\right)+\;Current\;Loss}\right)}\right] \end{array}$$

#### 3.4.2 Bollinger bands

The Bollinger Bands, developed by John Bollinger for technical analysis, are a volatility-dependent indicator consisting of three lines: a middle line (typically the moving average) and two bands on either side spaced equally apart. These bands may expand or narrow depending on the degree of market volatility. The mathematical expression for Bollinger Bands is as follows (Appel, 1985):


$$\begin{array}{l} Upper\;Band\;=\;Middle\;Band\;+\;\left(Multiplier\;*\;Standard\;Deviation\right) \end{array}$$

$$\begin{array}{l} Lower\;Band\;=\;Middle\;Band-\;\left(Multiplier\;*\;Standard\;Deviation\right) \end{array}$$

The upper band inside the Bollinger Bands framework signifies the upper limit of the price range. Calculating the Bollinger Bands begins by adding a particular multiple of the standard deviation to locate the center band. From there, it is designated as the lower limit in this framework, whichever minimum price falls within this interval. It is achieved by subtracting from its constant multiples of that standard deviation during calculation processing to determine these bands' values fully.

The intermediate strip indicates a separate scope or classification within the framework. Its location, flanked by two mobile averages, is a conventional means to discern it. Mobile averages, categorized into basic and exponential types, are statistical computations that determine the mean value of an asset over a designated duration.

**Figure 3:**
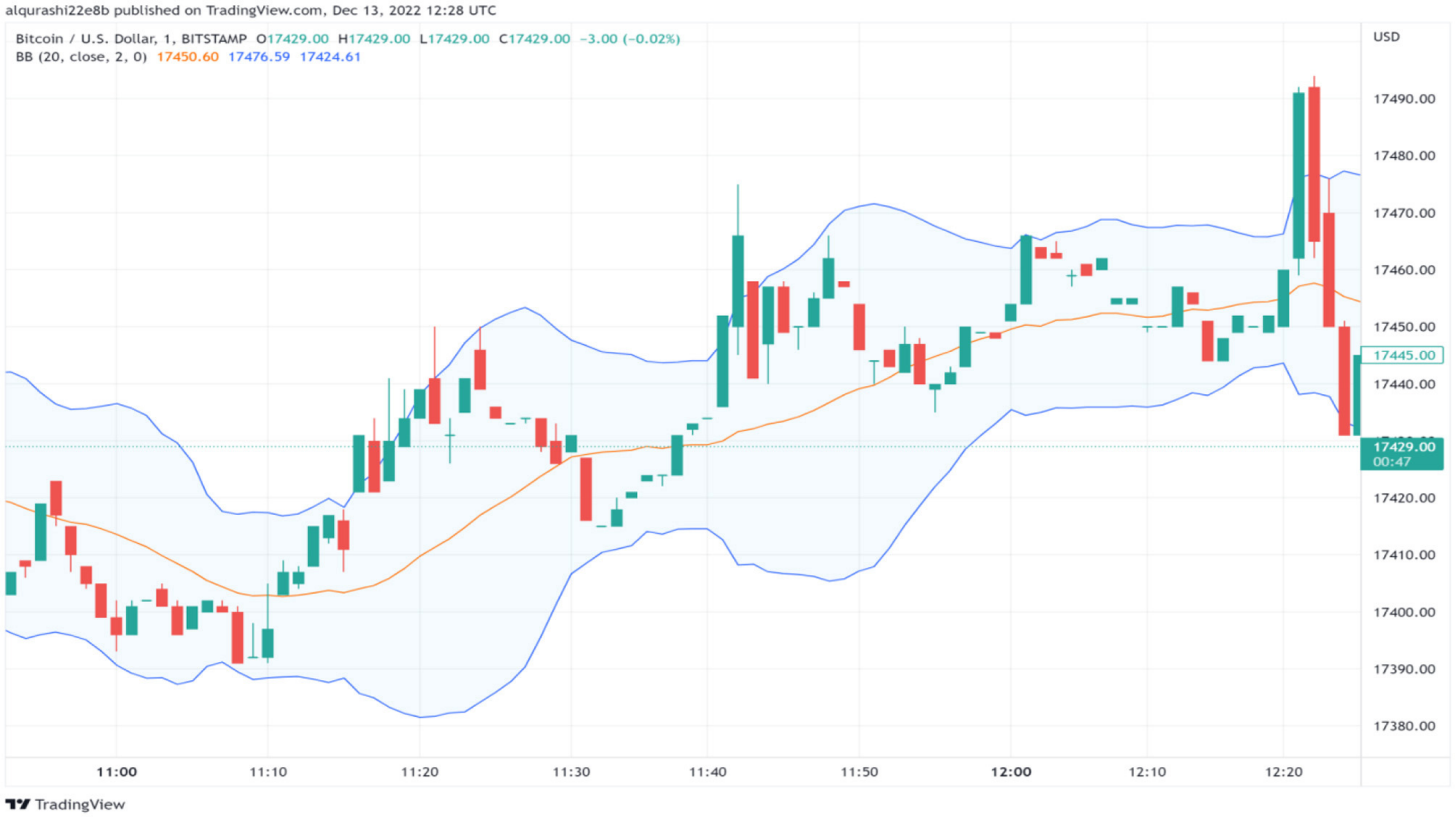
The period used here is 20, the default period in the Bollinger Bands indicator.

A “multiplier” is a mathematical idea used for multiplication and division. The constant value, typically denoted as 2, designates the quantity of standard deviations that should be added to the central band. The standard deviation is a statistical metric that measures how much-observed prices differ from the mean price, which indicates the central tendency. It assists in capturing market volatility. Bollinger Bands can help traders predict potential market reversals and measure the amount of price volatility. When the price exceeds the borders of the bands, it may suggest a decreasing strength in the existing trend, indicating the possibility of an oncoming reversal (illustrated in Figure 3).

The oversold condition happens when the cryptocurrency price passes under the lower band, while the overbought condition happens when the price passes higher than the upper band. Therefore, the buy signal occurs when the cryptocurrency price is oversold. The buy signal happens if the price < lower band.

#### 3.4.3 Moving average convergence divergence

The Moving Average Convergence Divergence (MACD) is a trend-following momentum indicator developed by Gerald Appel (Kang, 2021). This strategy comprises two moving averages: the MACD and signal lines. Moving Average Convergence Divergence (MACD) is the difference between a shorter-term exponential moving average, commonly spans 12 periods, and a more long-term exponential moving average, typically spans 26. A 9-period exponentially moving average of the Moving Average Convergence Divergence (MACD) line is commonly used to depict the signal line (Gerritsen et al., 2020).

**Figure 4:**
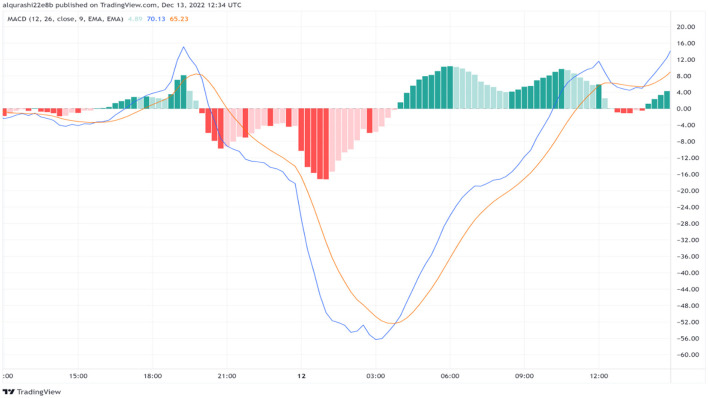
Resource for calculating the buy signals.

Traders actively seek instances of crossings occurring between the Moving Average Convergence Divergence (MACD) line and the signal line since these occurrences can indicate shifts in the direction of a given trend. Furthermore, the MACD histogram, which denotes the disparity between the MACD line and the signal line, furnishes insights into the prevailing trend's potency. In MACD, to create a buy signal, the MACD line value must become greater than the signal line value. The MACD line is usually presented as blue, and the signal line is orange. A buy signal is formed when the blue line cuts the orange line. In contrast, when the orange line (signal) cuts the blue line (MACD), a sell signal is formed (illustrated in Figure 4).

#### 3.4.4 Buy signal

A buy signal (Sadorsky, 2021a) is derived from technical analysis that indicates a potentially advantageous moment to acquire a financial item, such as a stock or currency pair. Purchase indications are often produced by indicators or patterns, such as the ones above (RSI, Bollinger Bands, MACD). The signs above may not provide absolute assurances of achieving success but rather function as indicators that suggest market circumstances may be converging in a manner conducive to a positive trajectory. Recognizing that these indicators are only tools in a trader's arsenal is critical. Properly using these indicators necessitates thoroughly evaluating various factors, including market context, volume, and extensive trend analysis.

### 3.5 Bagged tree model

Bootstrap Aggregating, also known as “bagging,” is an ensemble approach that involves training multiple decision tree models on various subsets of the provided data. By merging these models' predictions, overall accuracy improves while reducing overfitting risk. To execute this process, countless bootstrap samples must be generated from the given data before different decision trees are trained for each sample set. Next, forecasts produced by all such trees should combine to bolster model robustness and predictive skills. Consequently, bootstrap resampling used here produces varied training sets useful in developing a group or “ensemble” of decision trees; merge ensures variance reduction while promoting generalization further refined via repeated experimentation (Marti, 2023).

**Figure 5:**
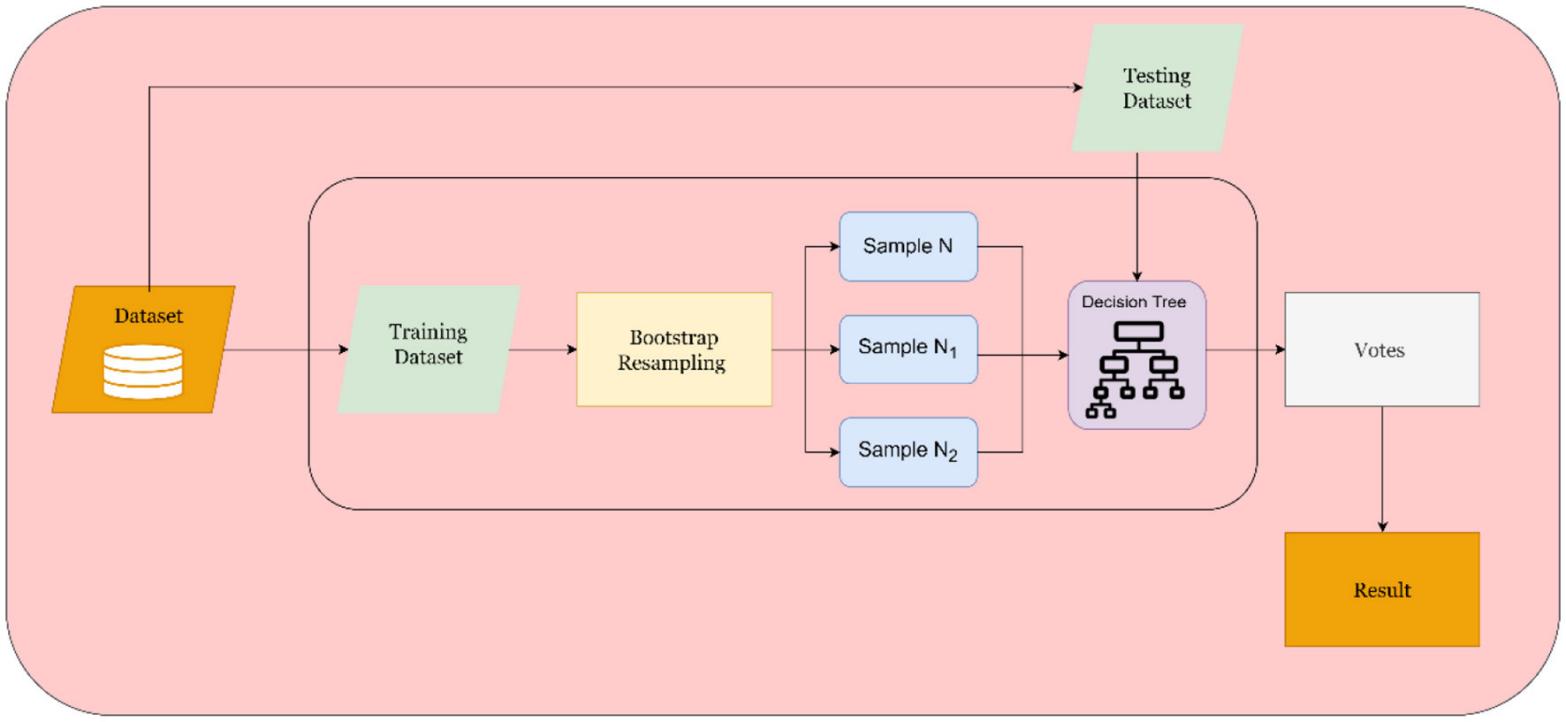
Structure of the BT model.

Figure 5 depicts the BT model method commences by randomly generating bootstrap replicates of the training dataset. This step divides the testing data into subsamples, and each sample serves as test data to generate a decision tree individually. Bootstrapping count decides the number of trees created. Finally, results from numerous samples are amalgamated using an overall voting scheme for better accuracy since more significant votes yield improved outcomes.

## 4 Discussion and result

This research exploits the BT model to predict buy signals of the Relative Strength Index (RSI), Bollinger Band (BB), and Moving Average Convergence/Divergence (MACD). The dataset employed in this research was collected from four different cryptocurrency platforms: Ethereum (ETH), Cardano (ADA), Binance Coin (BNB), and Bitcoin (BTC). As mentioned in the previous section, the dataset comprises sixteen features and three labels. The BT model was trained with the same dataset three times to predict each buy signal. The first training is performed to predict the RSI value. Secondly, the BB value is predicted, and the MACD value is predicted in the third training. The justification for undertaking three distinct training experiments is to analyze the result of each buy signal separately and select the outperforming buy signal among the three.

**Figure 6:**
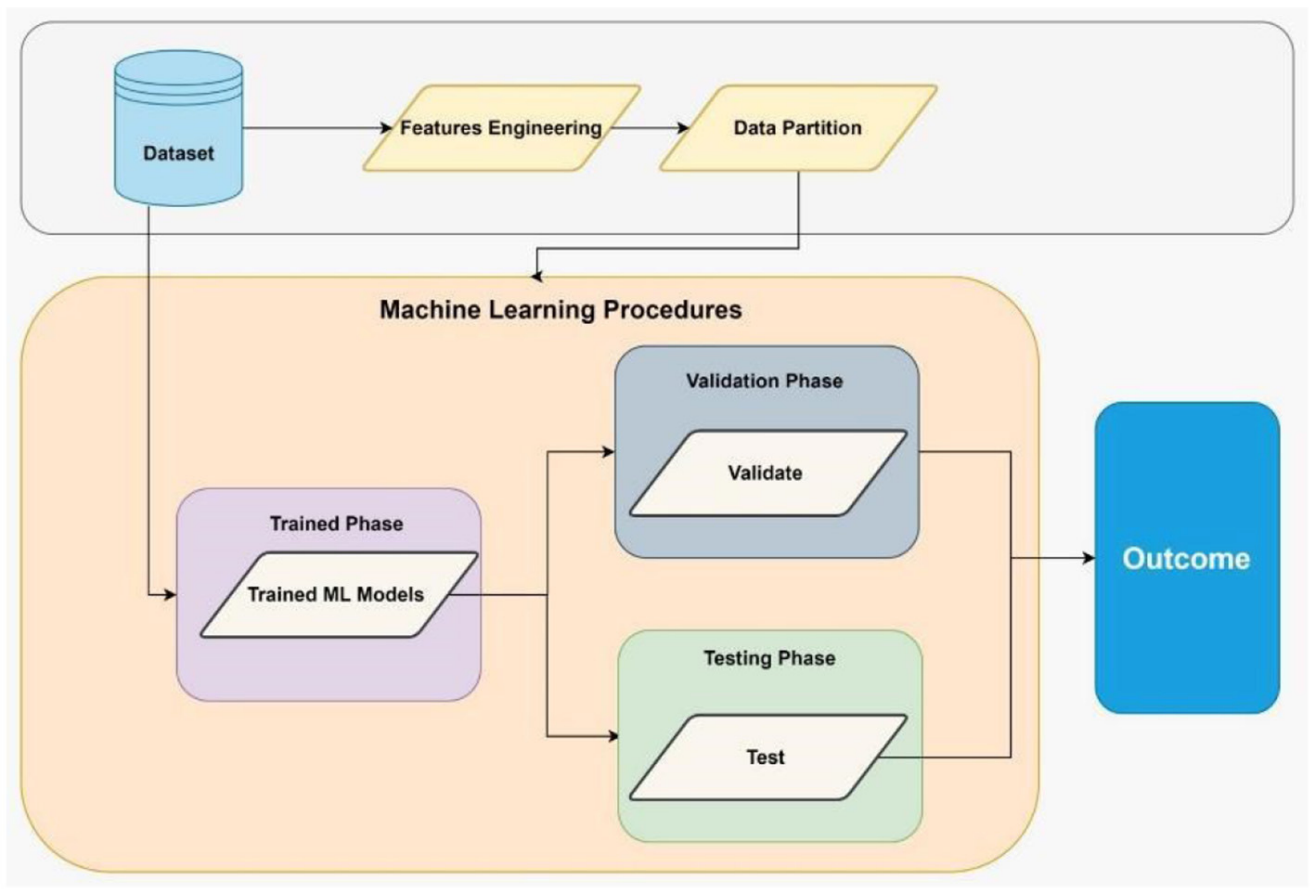
Experimental phases of machine learning.

Figure 6 illustrates the experiment phases of the machine learning model, including the BT model. The first step in machine learning data preparation involves applying feature engineering procedures. Feature engineering refers to selecting, modifying, and extracting unprocessed data to generate the variables needed for the goal of study or predicted modeling (Alsulami et al., 2022). What comes next involves the process of data partitioning, which is vital for ensuring the success of machine learning models. Data partitioning (Sadorsky, 2021b) divides a carefully selected dataset into multiple subsets, training, validation, and test sets.

For each training set, 80% of the dataset was preserved, and the remaining 20% was held for testing. The size of the training matrix is 335662 × 16, and the size of the test matrix is 83916 × 16. The BT model used a decision tree as a leaner type, and the maximum number of splits was 335662 with 30 learners. The maximum number of splits in the training can reach up to 335,662 because, in a bagged tree, the default maximum number of splits reaches the training data size in MATLAB's Classification Learner app. Each buy signal contains two possible values: 0 indicates “not to buy” (hold your decision), and 1 indicates “buy.” The evaluation metrics were computed for each buy signal to assess the performance of the BT model. The classifier accuracy is calculated using the following formula:


$$\begin{array}{l} ClassifierAccuracy\;=\frac{TP+TN}{TP+FP+TN+FN}\;\times \;100\;Where \end{array}$$

- TP is the true positive sample.
- TN true negative sample.
- FP is the false positive sample.
- FN is the false negative sample.

The precision which measures the ability of a classifier to predict positive instances is calculated as follows:


$$\begin{array}{l} Precision=\frac{TP}{TP+FP}\;\times \;100 \end{array}$$

The recall, also called sensitivity, is calculated as follows:


$$\begin{array}{l} Recall/sensitivity=\frac{TP}{TP+FN}\;\times \;100 \end{array}$$

The specificity which measures the accuracy of a classifier for the prediction of negative instances, is calculated as follows:


$$\begin{array}{l} Specificity=\frac{TN}{TN+FP}\;\times \;100 \end{array}$$

F1 score is the harmonic mean of precision formula and recall formula, and it is a specifically useful metric for measuring the accuracy of a classifier trained with an imbalanced dataset. The F1 score is computed as follows:


$$\begin{array}{l} {\text{F}}_{1}score=\frac{TP}{TP+\frac{FP+FN}{2}}\;\times \;100 \end{array}$$

Table 4 lists the results of each evaluation matric for each buy signal. RSI_buy_signal scores 100 % with each metric because its rules produced, as shown in Table 2 the lowest percentage, 3.61 %, compared to other buy signals. BB_buy_signal performed the lowest among the three buy signals since it is highly sensitive to the price. Nevertheless, the three buy signals are important in informing and shaping decision-making.

**Table 4:** Evaluation metrics of the BT model using test data.

**Evaluation metric**	**MACD_buy _signal**	**BB_buy _signal**	**RSI_buy _signal**
Classifier accuracy	96.00%	89.34%	100%
Precision	96.04%	68.70%	100%
Recall/ sensitivity	95.91%	50.56%	100%
Specificity	96.09%	96.03%	100%
F1-score	95.97%	58.25%	100%

Furthermore, 10-fold cross-validation is calculated to demonstrate the mitigation of overfitting and the generality of the BT model in predicting MACD_buy_signal, BB_buy_signal, and RSI_buy_signal. Looking at Table 5, we can observe that the classification accuracy, precision, recall, specificity, and F1-score of each buy_signal are consistent with the results presented in Table 4, confirming the validity of the experimental results performed in this study.

**Table 5:** Evaluation metrics of the BT model using 10-fold cross-validation.

**Evaluation metric**	**MACD_buy _signal**	**BB_buy _signal**	**RSI_buy _signal**
Classifier accuracy	95.86%	89.39%	100%
Precision	96.00%	68.89%	100%
Recall/ sensitivity	95.65%	51.00%	100%
Specificity	96.06%	96.05%	100%
F1-score	95.82%	58.61%	100%

In addition, we employed robust experimental methods using state-of-the-art machine-learning models to compare the response of the BT model. Four popular machine learning models were utilized: decision tree (DT), *k*-nearest neighbor (KNN), random forest (RF), and neural network (NN). The reason for conducting such experiments is to validate the performance of our proposed model, BT. Tables 6–8 comprehensively evaluate several machine-learning models against MACD_buy_signal, BB_buy_signal, and RSI_buy_signal. Each buy signal was evaluated using classifier accuracy, precision, recall/sensitivity, specificity, and F1-score.

**Table 6:** Evaluation metrics of ML models based on MACD_buy_signal.

**ML model**	**MACD_buy_signal**				
	**Accuracy**	**Precision**	**Recall/Sensitivity**	**Specificity**	**F1-score**
DT	92.63%	92.77%	92.38%	92.88%	92.57%
KNN	78.54%	78.26%	78.66%	78.41%	78.46%
NN	86.30%	86.65%	85.62%	86.97%	86.13%
RF	95.73%	95.69%	96%	95.73%	95.84%
**BT (proposed)**	**96.00%**	**96.04%**	**95.91%**	**96.09%**	**95.97%**

Bold values indicate the results obtained from our proposed Bagged Tree (BT) Model.

Regarding the MACD_buy_signal results presented in Table 6, the DT records an accuracy of 92.63%. However, the KNN and NN models score accuracies of 78.54% and 86.30%, respectively. Meanwhile, RF and our proposed model (BT) demonstrate a comparable performance, with a slight improvement noted in BT. Also, we can observe that the precision, recall, specificity, and F1-score metrics align closely with the accuracy figures for each model under MACD_buy_signal.

Shifting our focus to the RSI_buy_signal shown in Table 7, the impeccable performance of the DT, RF, and BTT models can be noticed. The KNN model also produces a respectable accuracy of 99.67%, but its recall and F1 score are slightly better than its precision. The NN scores an accuracy of 99.99%, with very close precision, recall, specificity, and F1 score values. Table 8 lists the performance of machine learning models against the BB_buy_signal. The DT model, which records an accuracy of 87.66%, faces a noticeable divergence between its precision and recall, causing an F1-score of 45.26%. The same applies to the KNN, neural network, RF, and BT models. It has also been observed that RF shows marginally better performance compared to our proposed model in predicting BB_buy_signal. BT and RF techniques employ decision trees to predict outcomes from provided data. These methods involve constructing multiple trees, each trained on a slightly different subset of the data, and then aggregating their predictions to improve accuracy (Marti, 2023). In BT, all available features are used in each tree, potentially leading to correlated trees. The final prediction in BT is derived by averaging the outcomes of all trees. In contrast, RF enhances diversity by randomly selecting a subset of features for each split within the trees, thus reducing correlation among them. A majority vote from all trees determines the final prediction in RF. Thus, each method adopts a distinct approach to predicting outcomes, leveraging ensemble strategies to mitigate the limitations of individual decision trees. Consequently, we chose BT as our main model to apply all dataset features.

**Table 7:** Evaluation metrics of ML models based on RSI_buy_signal.

**ML model**	**RSI_buy_signal**				
	**Accuracy**	**Precision**	**Recall/Sensitivity**	**Specificity**	**F1-score**
DT	100%	100%	100%	100%	100%
KNN	99.67%	95.33%	95.52%	99.82%	95.42%
NN	99.99%	99.93%	99.84%	99.99%	99.88%
RF	100%	100%	100%	100%	100%
**BT (proposed)**	**100%**	**100%**	**100%**	**100%**	**100%**

Bold values indicate the results obtained from our proposed Bagged Tree (BT) Model.

**Table 8:** Evaluation metrics of ML models based on BB_buy_signal.

**ML model**	**BB_buy_signal**				
	**Accuracy**	**Precision**	**Recall/Sensitivity**	**Specificity**	**F1-score**
DT	87.66%	65.06%	34.70%	96.79%	45.26%
KNN	84.60%	47.61%	47.35%	91.02%	47.48%
NN	89.33%	**71.27%**	45.98%	**96.81%**	55.89%
RF	**90.04%**	71.14%	**54%**	96.24%	**61.40%**
**BT (proposed)**	**89.39%**	**68.89%**	**51%**	**96.05%**	**58.61%**

Bold values indicate the results obtained from our proposed Bagged Tree (BT) Model.

Based on the experimental results, evaluations, and previous discussions, we conclude that the BT model provides promising results in predicting buy signal values; therefore, we confirm its vitality as a predictor for the buy signal for the three buy signals in this study.

## 5 Conclusion

This research studied the efficacy of MACD, RSI, and BB buy signals on purchase decisions. First, the cryptocurrency data was collected from Binance exchanges, including Bitcoin, Ethereum, Cardano (ADA), and Binance Coin (BNB). Subsequently, the dataset contained the technical indicators derived from the raw data. According to the study's findings, predicting buy signal values using a BT model provides promising results. Because the model can handle extreme volatility and unusual market patterns with ease, it gives investors useful information that they can use to make better decisions.

Several interesting paths for further study could be accomplished through several promising future research directions. First, making the model more accurate and versatile in its predictions is to add more features to it. The other approach can be investigating the sell signal to adapt to various market conditions. Lastly, the model's ability to deal with complex market dynamics could be even better if reinforcement learning is to be implanted. By following these research directions, we can significantly improve the reliability and capacity of our model, thereby turning it into a more effective tool for cryptocurrency investors.

## Data availability statement

The original contributions presented in the study are included in the article/supplementary material, further inquiries can be directed to the corresponding author.

## Author contributions

RA: Writing – original draft, Writing – review & editing. QA: Writing – original draft, Writing – review & editing. AAls: Writing – original draft, Writing – review & editing. BA: Writing – original draft, Writing – review & editing. AAlqu: Writing – original draft, Writing – review & editing. MM: Writing – original draft, Writing – review & editing. AAlqa: Writing – original draft, Writing – review & editing. NA: Writing – original draft, Writing – review & editing.

## References

[B1] Alsulami A. A. Abu Al-Haija Q. Tayeb A. Alqahtani A. (2022). An intrusion detection and classification system for IoT traffic with improved data engineering. Appl. Sci. 12:12336. 10.3390/app122312336

[B2] Anghel D. G. (2021). A reality check on trading rule performance in cryptocurrency: machine learning vs. technical analysis. Financ. Res. Lett. 39:101655. 10.1016/j.frl.2020.101655

[B3] Appel G. (1985). The Moving Average Convergence-Divergence Trading Method: Advanced Version. Scientific Investment Systems.

[B4] Arowolo M. O. Ayegba P. Yusuff S. R. Misra S. (2022). A Prediction Model for Bitcoin Cryptocurrency Prices. Blockchain Applications in the Smart Era. Cham: Springer International Publishing, 127–146.

[B5] Asgari M. Khasteh H. (2021). Profitable strategy design for trades on cryptocurrency markets with machine learning techniques. arXiv preprint arXiv:2105.06827.

[B6] Bello A. S. Schneider J. Di Pietro R. (2023). “LLD: a low latency detection solution to thwart cryptocurrency pump and dumps,” in 2023 IEEE International Conference on Blockchain and Cryptocurrency (ICBC) (IEEE), 1–9.

[B7] Bellocca G. P. Attanasio G. Cagliero L. Fior J. (2022). Leveraging the momentum effect in machine learning-based cryptocurrency trading. Mach. Learning Appl. 8:100310. 10.1016/j.mlwa.2022.100310

[B8] Binance Exchange (2018). Binance Whitepaper V1.1. Available online at: https://whitepaper.io/document/10/binance-whitepaper (accessed January 14, 2023).

[B9] BTC/USDT (2022). Available online at: https://www.binance.com/en/trade/btc_USDT?theme=darkandtype=spot (accessed December 24, 2022).

[B10] Busayatananphon C. Boonchieng E. (2022). “Financial technology DeFi protocol: a review,” in 2022 Joint International Conference on Digital Arts, Media, and Technology with ECTI Northern Section Conference on Electrical, Electronics, Computer and Telecommunications Engineering (ECTI DAMT and NCON) (IEEE), 267–272.

[B11] CCXT/CCXT (n.d.). A JavaScript / Typescript / Python / C# / PHP Cryptocurrency Trading API With Support for More Than 100 Bitcoin/Altcoin Exchanges GitHub. Available online at: https://github.com/ccxt/ccxt (accessed January 11, 2024).

[B12] Çelik O. (2019). Implementation of Technical Analysis on Selected Cryptocurrencies (Doctoral dissertation). Istanbul: Marmara Universitesi.

[B13] Cohen G. (2022). Algorithmic trading and financial forecasting using advanced artificial intelligence methodologies. Mathematics 10:3302. 10.3390/math10183302

[B14] Cohen G. Qadan M. (2022). The complexity of cryptocurrencies algorithmic trading. Mathematics 10:2037. 10.3390/math10122037

[B15] Cryptocurrency Exchange for Bitcoin Ethereum, Altcoins, and Binance. (2023). Available online at: https://www.binance.com/en (accessed August 9, 2023).

[B16] Cunha R. Sebastião H. (2021). From Bitcoin to central bank digital currencies: making sense of the digital money revolution. Fut. Int. 13:165. 10.3390/fi13070165

[B17] Detzel A. Liu H. Strauss J. Zhou G. Zhu Y. (2021). Learning and predictability via technical analysis: evidence from bitcoin and stocks with hard-to-value fundamentals. Financ. Manage. 50, 107–137. 10.1111/fima.12310

[B18] Dolatsara H. A. Kibis E. Caglar M. Simsek S. Dag A. Dolatsara G. A.. (2022). An interpretable decision support systems for daily cryptocurrency trading. Exp. Syst. Appl. 203:117409. 10.1016/j.eswa.2022.117409

[B19] Fang F. Chung W. Ventre C. Basios M. Kanthan L. Li L.. (2021). Ascertaining price formation in cryptocurrency markets with machine learning. The Eur. J. Financ. 22, 1–23. 10.1080/1351847X.2021.1908390

[B20] Fang F. Ventre C. Basios M. Kanthan L. Martinez-Rego D. Wu F.. (2022). Cryptocurrency trading: a comprehensive survey. Financ. Innov. 8, 1–59. 10.1186/s40854-021-00321-638111683

[B21] Gerritsen D. F. Bouri E. Ramezanifar E. Roubaud D. (2020). The profitability of technical trading rules in the Bitcoin market. Financ. Res. Let. 34:101263. 10.1016/j.frl.2019.08.01137885713

[B22] Gurrib I. Kamalov F. (2019). The implementation of an adjusted relative strength index model in foreign currency and energy markets of emerging and developed economies. Macroecon. Financ. Emerg. Market Econ. 12, 105–123. 10.1080/17520843.2019.1574852

[B23] Hairudin A. Sifat I. M. Mohamad A. Yusof Y. (2022). Cryptocurrencies: a survey on acceptance, governance, and market dynamics. Int. J. Financ. Econ. 27, 4633–4659. 10.1002/ijfe.239234778834

[B24] Hansen S. Wicaksana A. Khaliq A. Q. (2022). Multivariate cryptocurrency prediction: Comparative analysis of three recurrent neural networks approaches. J. Big Data 9:1. 10.1186/s40537-022-00601-7

[B25] Hu S. Zhang Z. Lu S. He B. Li Z. (2023). Sequence-based target coin prediction for cryptocurrency pump-and-dump. Proc. ACM Manag. Data 1, 1–19. 10.1145/3588686

[B26] Iqbal M. Iqbal M. Jaskani F. Iqbal K. Hassan A. (2021). Time-series prediction of the cryptocurrency market using machine learning techniques. EAI Endors. Trans. Creat. Technol. 8:28. 10.4108/eai.7-7-2021.170286

[B27] Jaquart P. Dann D. Weinhardt C. (2021). Short-term bitcoin market prediction via machine learning. The J. Financ. Data Sci. 7, 45–66. 10.1016/j.jfds.2021.03.001

[B28] Jiang P. Liu Z. Abedin M. Z. Wang J. Yang W. Dong Q.. (2024). Profit-driven weighted classifier with interpretable ability for customer churn prediction. Omega 125:103034. 10.1016/j.omega.2024.103034

[B29] Joiner D. Vezeau A. Wong A. Hains G. Khmelevsky Y. (2022). “Algorithmic trading and short-term forecast for financial time series with machine learning models; state of the art and perspectives,” in 2022 IEEE International Conference on Recent Advances in Systems Science and Engineering (RASSE) (IEEE), 1–9. 10.1109/RASSE54974.2022.9989592

[B30] Kamara A. F. Chen E. Pan Z. (2022). An ensemble of a boosted hybrid of deep learning models and technical analysis for forecasting stock prices. Inf. Sci. 594, 1–19. 10.1016/j.ins.2022.02.015

[B31] Kang B. K. (2021). Improving MACD technical analysis by optimizing parameters and modifying trading rules: evidence from the Japanese Nikkei 225 futures market. J. Risk Financ. Manage. 14:37. 10.3390/jrfm14010037

[B32] Kumar K. C. Rajesh M. (2022). “Ethereum and Binance price forecasting using machine learning,” in 2022 IEEE 3rd Global Conference for Advancement in Technology (GCAT). IEEE, 1–8.

[B33] La Morgia M. Mei A. Sassi F. Stefa J. (2023). The doge of wall street: Analysis and detection of pump and dump cryptocurrency manipulations. ACM Trans. Internet Technol. 23, 1–28. 10.1145/3561300

[B34] Lamothe-Fernández P. Alaminos D. Lamothe-López P. Fernández-Gámez M. A. (2020). Deep learning methods for modeling bitcoin price. Mathematics 8:1245. 10.3390/math8081245

[B35] Lauguico S. Concepcion I. I. Alejandrino R. Macasaet J. Tobias D. Bandala R. R.. (2019). “A fuzzy logic-based stock market trading algorithm using bollinger bands,” in 2019 IEEE 11th International Conference on Humanoid, Nanotechnology, Information Technology, Communication and Control, Environment, and Management (HNICEM) (IEEE), 1–6.

[B36] Lee J. Y. (2019). A decentralized token economy: how blockchain and cryptocurrency can revolutionize business. Bus. Horiz. 62, 773–784. 10.1016/j.bushor.2019.08.003

[B37] Liu Z. Jiang P. Wang J. Du Z. Niu X. Zhang L.. (2023). Hospitality order cancellation prediction from a profit-driven perspective. Int. J. Contemp. Hosp. Manage. 35, 2084–2112. 10.1108/IJCHM-06-2022-0737

[B38] Marti G. (2023). Decoding the Quant Market: A Guide to Machine Learning in Trading. 10.2139/ssrn.4422374

[B39] Matytsin D. E. (2021). Internet Investing as a Remote Algorithm of the Retail Investment Financing. Cham: Springer International Publishing, 1850–1857.

[B40] Nakamoto S. (2017). Bitcoin: A Peer-to-Peer Electronic Cash System. Available online at: http://www.bitcoin.org/bitcoin.pdf (accessed October 10, 2008).

[B41] Namasudra S. Deka G. C. Johri P. Hosseinpour M. Gandomi A. H. (2021). The revolution of blockchain: State-of-the-art and research challenges. Arch. Comput. Methods Eng. 28, 1497–1515. 10.1007/s11831-020-09426-0

[B42] Patel M. M. Tanwar S. Gupta R. Kumar N. (2020). A deep learning-based cryptocurrency price prediction scheme for financial institutions. J. Inf. Secur. Appl. 55:102583. 10.1016/j.jisa.2020.102583

[B43] Sadorsky P. (2021a). A random forests approach to predicting clean energy stock prices. J. Risk Financ. Manage. 14:48. 10.3390/jrfm14020048

[B44] Sadorsky P. (2021b). Predicting gold and silver price direction using tree-based classifiers. J. Risk Financ. Manage. 14:198. 10.3390/jrfm14050198

[B45] Saxena S. Vyas S. Kumar B. S. Gupta S. (2019). “Survey on online electronic paymentss security,” in 2019 Amity International Conference on Artificial Intelligence (AICAI) (IEEE), 756–751.

[B46] Senthilkumar D. (2021). Data confidentiality, integrity, and authentication. Research Anthology on Blockchain Technology in Business, Healthcare, Education, and Government. London: IGI Global.

[B47] Shah D. Isah H. Zulkernine F. (2019). Stock market analysis: a review and taxonomy of prediction techniques. Int. J. Finan. Stu. 7:26. 10.3390/ijfs7020026

[B48] Shynkevich A. (2021). Impact of Bitcoin futures on the informational efficiency of the Bitcoin spot market. J. Fut. Markets 41, 115–134. 10.1002/fut.22164

[B49] Smales L. A. (2022). Investor attention in cryptocurrency markets. Int. Rev. Financ. Anal. 79:101972. 10.1016/j.irfa.2021.101972

[B50] Sun T. Yu W. (2020). A formal verification framework for security issues of blockchain smart contracts. Electronics 9:255. 10.3390/electronics9020255

[B51] Ta-lib/Ta-lib-Python (n.d.). Python Wrapper for Ta-Lib (http://ta-lib.org/) GitHub. Available online at: https://github.com/TA-Lib/ta-lib-python (accessed January 11, 2024).

[B52] Toledo J. D. M. Souza D. Y. (2022). Signal Prediction in Cryptocurrency Tradeoperations: A Machine Learning-Based Approach. Available online at: https://ssrn.com/abstract=4062476

[B53] Vachhani H. Obiadat M. S. Thakkar A. Shah V. Sojitra R. Bhatia J.. (2020). “Machine learning based stock market analysis: a short survey,” in International Conference on Innovative Data Communication Technologies and Application (Cham: Springer), 12–26.

[B54] Vo A. Yost-Bremm C. (2018). A high-frequency algorithmic trading strategy for cryptocurrency. J. Comput. Inf. Syst. 60, 555–568. 10.1080/08874417.2018.155209036938429

[B55] Vo A. D. Nguyen Q. P. Ock C. Y. (2019). Sentiment analysis of news for effective cryptocurrency price prediction. Int. J. Know. Eng. 5, 47–52. 10.18178/ijke.2019.5.2.116

[B56] Wilder J. W. (1978). New Concepts in Technical Trading Systems. Greensboro, NC.

[B57] Yu S. E. (2022). On methods of building the trading strategies in the cryptocurrency markets. Discr. Contin. Models Appl. Comput. Sci. 30, 79–87. 10.22363/2658-4670-2022-30-1-79-87

